# Health-promoting behaviors and intermediary social determinants of health in low and high-risk pregnant women: an unmatched case-control study

**DOI:** 10.1186/s12884-022-04784-y

**Published:** 2022-05-28

**Authors:** Marzieh Masjoudi, Somayyeh Khazaeian, Sanna Malekzadeh, Azita Fathnezhad-Kazemi

**Affiliations:** 1 Department of Midwifery, Faculty of Nursing and Midwifery Islamic Azad University, Rasht branch, Rasht, Iran; 2 Pregnancy Health Center, Department of Midwifery, Zahedan University of Medical Sciences, Zahedan, Iran; 3 Department of Midwifery, Faculty of Nursing and Midwifery, Tabriz Medical sciences, Islamic Azad University, Tabriz, Iran; 4 Women’s Reproductive and Mental Health Research center, Tabriz Medical Sciences, Islamic Azad University, Tabriz, Iran

**Keywords:** Health-promotion, Social support, Gestational hypertension, Stress, Self-Efficacy, High-risk

## Abstract

**Background:**

High-risk pregnancies require increased health and care resources to reduce the severe perinatal consequences. The adoption of a health-promoting lifestyle and social determinants is an important strategy for achieving the desired outcomes of pregnancy. This study aimed to compare intermediate determinants of social health in low and high-risk pregnant women.

**Methods:**

This unmatched case-control study was performed with a ratio of 1: 2 and 300 pregnant women including 200 healthy and 100 pregnant women with gestational hypertension were included using the available sampling technique. Data were collected using socio-demographic and obstetrics, Health-promoting behaviors, Self-efficacy, Perceived stress, and Social support questionnaires by the self-report method.

**Results:**

There was no significant difference in the demographic characteristics between the two groups, except for the spouse's education status. The total score of health-promoting behaviors and social support in the healthy group was significantly higher than women with gestational hypertension. However, the perceived stress in women with gestational hypertension was significantly higher than in the healthy group. In the multivariate analysis, those women with high stress [AOR 1.13, 95% CI (1.08–1.18)] and whose Spouse’s Educational status was low [AOR 4.94, 95% CI (1.54–15.81)] had higher odds of gestational hypertension than women who haven’t respectively. The development of gestational hypertension was decreased by increasing the score of social support [AOR 0.96, 95% CI (0.93–0.98)]. The results showed that the two variables of social support (β=0.331) and self-efficacy (β=0.215) have the greatest impact on the score of health-promotion behaviors, respectively. Based on regression analysis, 21.2% of the health-promotion behaviors changes could be explained by three independent variables.

**Conclusion:**

Women with gestational hypertension have unhealthier lifestyles. Having a high level of stress is a risk factor for gestational hypertension but Social support has a protective effect on it. Recognizing the risk factors of gestational hypertension could help the determination of high-risk cases and it is important to pay attention to women's psychosocial to create appropriate sources of social support and provide the necessary action to reduce stress.

## Introduction

Mother’s lifestyle during pregnancy has long lasting effects on both mother and her child‘s health [1, 2]. Pregnancy is one of the most important and critical period of women’s life [3], because pregnancy related changes in women’s body makes her a person with new psychological and physical characteristics [4, 5]. Sometimes these changes are beyond her control [5, 6] and lead to physical and spiritual harms [5], in such a way that can affect the behavior and lifestyles of pregnant women [2, 7]. However, most pregnancies come to an end without difficulty, some women experience complications such as hypertension and diabetes, which put them into high-risk pregnancy category [8, 9]. It is reported that the prevalence of high-risk pregnancy is about 15-20 percent across the world [9]. But 50 percent of prenatal mortalities occur among high-risk pregnancies [10]. In addition to natural spiritual and psychological changes of pregnancy, concerns about fetus future increase in women with high-risk pregnancy [4, 9]. These women face changes in personal, family and social life that can affect their quality of life and thus impose health effects on the mother and the newborn [11]. So, one of the most important issues in maternal and child health topics is how to spend pregnancy well [2, 4]. Although most pregnant women eager to perform health promoting behaviors to improve the health condition of themselves and their newborn, but theoretically bringing up them as high risk group is considered as a threatening factor by women [1, 9, 10]. This perceived threat may enhance the stress level and influence their use of protective behaviors [12]; so that the use of health-promoting behaviors in high risk women are different from low-risk ones [8]. Identifying effective factors on health behaviors is helpful to women with high-risk pregnancies [13], and since present health perspective has focused on health determinants more, each of these determinants, by themselves or by affecting each other, strongly affects the state of health [14]. Based on the WHO conceptual framework of commission on social determinants included: 1. structural determinants in 2 categories: a) socioeconomic and political context such as economic processes, culture, and the function of social welfare system and b) others such as education, income, sex, race, ethnicity and employment status, which create different unequal socioeconomic groups and, ultimately, form the social class of a person; 2. Intermediary determinants of health including behaviors and psychosocial factors [15]. Social determinants of health, such as social support and perceived stress, affect individuals' health behaviors; and are an integral part of promoting health [16]. Stress as an intermediate factor in social determinants is associated with many diseases, including cardiovascular disease [17]; In contrast to stress, there is social support that reduces stress and increases adaptation. Studies show positive effect of social support on improving health status [18]. Bandura believes self–efficacy feelings enables people to perform extraordinary tasks using skills in dealing with obstacles; thus perceived self - efficacy is an important factor to successfully perform its function and essential skills to be carried out [19, 20] as well as Self-efficacy is one of essential tools for continuity of cares and fulfil health promoting behaviors to reach desired pregnancy and newborn outcomes [13]. Some studies indicate the adverse effects of psychosocial factors such as stress during pregnancy on adverse pregnancy outcomes [21]. Significant relation has been reported between social support and adoption of a healthy lifestyle [22]. Also, there is a negative relationship between social support and adverse pregnancy outcomes [18, 22]. It is believed that the social support is a kind of barrier against the stressors which may increase disease like hypertension [18]. Nevertheless, it is shown that the stress level is lower in women with high-risk pregnancy who participate in health promoting programs [23]. Thus, it is necessary to maintain and promote the health condition of women with high-risk pregnancy by creating appropriate intervention strategies on the basis of understanding affecting factors and their behaviors and psychosocial characteristics to ensure the health of this important group of society, so perhaps it reduced the burden of disease and maternal and neonatal mortality. The aim of this study was to compare social support, perceived stress, self-efficacy, as psychosocial factors and health-promoting behaviors in low and high-risk pregnant women in Iran.

## Methods

### Study design and participants

This unmatched case–control study was conducted on 300 pregnant women attending the antenatal care clinic of 29-Bahman Hospital during the first 6 months of 2021 in Tabriz, Iran. The inclusion criteria were: Iranian nationality, singleton pregnancy, ability to read and write in Persian, gestational age≥28 weeks, no experience of stressors in the last 6 months (declared with participants, like death of some family members because it may have other psychological effects), absence of any medical and obstetrics disease/disorder in the both groups, and presence of the pregnancy-induced hypertension (Blood pressure ≥140/90) just in the case group. The exclusion criteria were unwillingness to partake and failure to complete the questionnaire.

### Sample size and sampling

The power analysis method was used to calculate the sample size. Since the largest sample size was obtained by considering health-promoting behaviors, this result was applied to estimate the sample size. In this regard, considering the results of a study carried out by Kavlak et al., [24] and Malakouti et al., [2] the mean scores of health-promoting behaviors in healthy pregnant woman and women with preeclampsia were 2.57 (0.42) and 2.4 (0.4), respectively. The effect size was calculated as 0.42. However, the sample size was calculated as 90 for each group considering 80% test power and 0.05 Type I error. It should be noted that the G*Power software was exploited to calculate the sample size. We considered the sample size to be 10% larger than the sample size calculated to increase the study power, the final sample size was adjusted to 100. Also, considering the ratio of 1 to 2, the sample size for the control group (low-risk) was 200 people.

The Sampling was performed acquiring the ethics permit from the ethics committee of Islamic Azad University of Medical Sciences and obtaining permission from the authorities of the hospital. This study used convenience sampling for which the author visited the obstetrics clinic of the Hospital. Subsequently, all pregnant women over 28 weeks of gestation who met the inclusion criteria were registered and completed the questionnaires by self-report method. Before recruiting the participants, they were informed about the aims and method of study, their voluntary participation, confidentiality, privacy protection, and the participant’s right to quit the study at any stage of data collection. If participants had a problem understanding the questionnaire items while completing the questionnaire, they would be answered immediately by the author.

### Instruments

#### Socio-demographic and obstetrics characteristics

It consists of the socio-demographic variables of pregnant women including maternal and spousal age, academic level, occupational status of pregnant women and their spouses, self-assessment of household economic status, as well as obstetrics characteristics, including gestational age (measured according to first-trimester ultrasound), number of pregnancies and parity, as well as body mass index was calculated with self-reported weight and height measured at a first prenatal visit by using the formula weight/height^2^.

#### Health-promoting Lifestyle Profile-II Questionnaire

This questionnaire contains 52 items that are categorized into six domains: a: nutrition (nine items; choose a diet low in fat, saturate fat, and cholesterol), b: physical activity (eight items; exercise vigorously for 20 or more minutes at least three times a week), c: health responsibility (nine items; read or watch TV programs about improving health), d: stress management (eight items; take some time for relaxation each day), e: interpersonal and social relationships (nine items; maintain meaningful and fulfilling relationships with others), and f: spiritual growth (nine items; Feel I am growing and changing in positive ways). A four-point Likert scale was used to measure each behavior, with ranges of never (1), sometimes (2), frequently (3), and regularly (4). The total score for these behaviors is within the range of 52–208 [25]. According to the literature, a health-promoting lifestyle is a multi-dimensional pattern of self-initiated feelings and behaviors aiming at ensuring an individual’s health, self-actualization, and self-accomplishment [26, 27]. The validity and reliability of Iranian version of HPLP-II, on the population based study, has been evaluated, and the Cronbach's alpha coefficients for the total tool and its dimensions were obtained as 0.82 within the range of 0.64–0.91 [22], respectively. In addition, the questionnaire had sufficient stability (0.89) [28].

#### The multidimensional scale of perceived social support

The perceived social support questionnaire designed by Zimet et al., that encompasses 12 items scored based on a Likert scale [29]. The questionnaire evaluates three domains of a: perceived support from the family (four items; My family is willing to help me make decisions), b: perceived support from friends (four items; I have friends with whom I can share my joys and sorrows.), and c: significant other (four items; there is a special person who is around when I am in need). The items are scored based on a seven-point Likert scale from “completely disagree” (score: 1) to “completely agree” (degree:7) where the minimum and maximum scores are 12 and 84, respectively [30]. Perceived social support refers to how individuals perceive friends, family members and others as sources available to provide material, psychological and overall support during times of need [31]. The instrument validity and reliability were confirmed in Iran; its validity was confirmed through content analysis and reliability in various studies was established using Cronbache's alpha coefficient (α=0.86-0.9 for the subscales and 0.86 for the whole instrument [32].

#### Self-efficacy questionnaire

The self-efficacy was measured using the General Self-Efficacy Scale of Sherer and et al., [33]. This scale is consisted of 17 items (example of items include: “When I make plans, I am certain I can make them work”) scored based on a Likert-scale which was rated from completely disagree to completely agree and each item is scored from 1 to 5. Items 2, 4, 5, 6, 7, 10, 11, 12, 14, 15, 16, and 17 are scored inversely [34]. As a result, the maximum and minimum scores are 85 and 17, respectively. The validity and reliability of the questionnaire was approved in IRAN by Asgharnejad et al [35]. The instrument measures someone's self-belief in their ability to build a sense of personal strength as they apply it to their day-to-day life [36].

#### Perceived Stress Scale (PSS)

PSS is provided by Cohen et al. [37] in 1983 with 3 versions of 4, 10 and 14 that was applied for measuring perceived stress in past 1 month. It is a measure of the degree to which situations in one's life are appraised as stressful and the scale also includes a number of direct queries about current levels of experienced stress [37, 38]. We used version 14 in this study. Each question has 5 options that half of them are direct (0, 1, 2, 3 and 4), and the other half are reverse (4, 3, 2, 1 and 0) scoring formats. All items are based on the Likert scale (0=never, 1=low, 2=moderate, 3=much and 4=very much) scoring. Scores are ranged between 0–56 sets. It should be noted that 7 questions as positive concepts (4, 5, 6, 7, 9, 10 and 13) are reverse-scored (4=never, 3=little, 2=moderate, 1=much, and 0= very much) [39, 40]. The homogeneity coefficients of this questionnaire in the Iranian population were also confirmed by Harris and Mousavi with the Cronbach's alpha being 0.84 [41]. High perceived stress was defined as a PSS score <30 [42].

#### Analysis

Data analysis was performed in SPSS software (version 22) Scoring of sociodemographic, health-promoting behaviors, social support, **s**elf-efficacy and Perceived Stress were described by frequency (percent), as well as mean (Standard Deviation). The association between variables were determined using the t-test, chi-square, Kruskal-Wallis, Mann-Whitney U, and Spearman's correlation. Multiple logistic regression (inter method) analyses were used to indicate the association between the dependent (with hypertension vs. without hypertension) and independent variables. Then, independent variables, with *P* ≤ 0.05 on bivariate tests inserted into the multivariate linear regression model (enter method). The normality of quantitative data was measured based on Kolmogorov–Smirnov test. Since the total score of health-promotion behavior was not normally distributed, this value was first converted by using a natural logarithm (Ln) transformation which yielded distributions that did not significantly deviate from normality then It was used in linear regression. All the statistical tests were two-sided, using a significance level of *p* < .05.

## Results

### Demographic Variables

A total of 300 pregnant women (200 healthy pregnant women and 100 pregnant women with gestational hypertension) participated in this study. There was no significant difference in the age of pregnant women in the two groups of healthy and gestational hypertension (28.67 vs 27.71, respectively). Most women in both groups were in the age group of 20-30 years and most of them in the case and control groups were multiparous (64% vs 60.5%), had a diploma (28.3% vs 38.0% respectively) and were housewife (89% vs 93.5%). There was no significant difference in the demographic characteristics between the two groups, except the spouse's education status. Also, there was a significant difference in gestational age between case and control groups [34.33 (3.90) vs 37.10 (5.05), *P* <0.001] Table 1.

**Table 1:** Demographic and obstetrics characteristics and their relationship with health-promoting lifestyle profile in two groups

Variable		All ***n***= 300	Pregnancy with induced hypertension ***n*** =100	Healthy pregnant women ***n***= 200	***P***-value	HPLP ***P***-value
**Mother’s Age (years)**	**Mean(SD)**	28.03 (5.16)	28.67 (6.42)	27.71(4.4.38)	0.702^a^	0.201^c^
>20	**n(%)**	28(9.3)	12(12.0)	16 (8.0)		
20-30		178 (59.3)	56 (56.0)	122(61.0)		
30-40		94 (31.3)	32 (32.0)	62(31.0)		
**Spouse’s Age (year)**	**Mean(SD)**	33.07 (6.00)	33.69(7.74)	32.76(4.89)	0.847^a^	0.258^c^
20-30	**n(%)**	101(33.7)	35(35.0)	66(33.2)		
30-40		167(55.7)	47 (47.0)	120(60.3)		
40-50		25(8.3)	11 (11.0)	13(6.5)		
>50		7(2.3)	7(7.0)	0(0)		
**Mother’s Educational status**	**n (%)**					
Primary school		12(4.0)	7(2.3)	5(1.7)	0.088^b^	0.003^c*^
Secondary school		56(18.7)	19(6.3)	37(12.3)		
Diploma		159(53.0)	45(28.3)	114(38.0)		
University		73(24.3)	29(9.7)	44(14.7)		
**Mother’s Employment status**	**n (%)**					
Housewife		276(92.0)	89(89.0)	187(93.5)	0.182^b^	0.094^d^
Employed		24(8.0)	11(11.0)	13(6.5)		
**Spouse’s Educational status**	**n(%)**					
Primary school		22(7.3)	11(11.0)	11(5.5)	0.007^b^*	0.832^c^
Secondary school		88(29.3)	33(33.0)	55(27.5)		
Diploma		117(39.0)	43(43.0)	74(37.0)		
University		73(24.3)	13(13.0)	60(30.0)		
**Spouse’s Employment status**	**n(%)**					
Worker		58(19.3)	15(15.0)	43(21.5)	0.246^b^	0.0843^d^
Employed		39(13.0)	11(11.0)	28(14.0)		
Self-employed		203(67.7)	74(74.0)	129(64.5)		
**Housing**						
Yes		148 (49.3)	41 (41)	107 (53.5)	0.041	0.249
No		152 (50.7)	59 (59)	93 (46.5)		
**Household income**						
< 5 million Rials		250 (83.3)	80 (80)	170 (85)	0.273	0727
≥ 5 million Rials		50 (16.7)	20 (20)	30 (15)		
**Parity**	**n(%)**					
Nulliparous		115(38.3)	36(36.0)	79(39.5)	0.557^b^	0.971^d^
Parous		185(61.7)	64(64.0)	121(60.5)		
**BMI**	**Mean(SD)**	25.62(4.23)	25.71(4.26)	25.44(4.20)	0.556^a^	0.002^c*^
Underweight	**n(%)**	23 (7.7)	7(7.0)	16(8.0)		
normal		121(40.3)	46(46.0)	75(37.5)		
Overweight		112(37.3)	33(33.0)	79(39.5)		
Obese		44(14.7)	14(14.0)	30(15.0)		

^a^t-test, ^b^ chi-square, ^c^ Kruskal-Wallis, ^d^ Mann-Whitney U, ^*^ statistically significant

Preliminary analysis showed that only statistically significant differences were observed between different levels of education of pregnant women and body mass index with health-promoting behaviors. So that the mean scores of health-promoting behaviors in participants with diploma and university education were higher than illiterate ones. Also, the total score of health-promoting behaviors in obese and overweight women was lower than normal weight Table 1.

### Comparison of health-promoting behaviors between two groups

The total score of health-promoting behaviors in the healthy group was significantly higher than

women with gestational hypertension [134.15 (19.03) vs 129.83 (15.04), *P* = 0.049]. Among the various dimensions of health-promoting behaviors in the healthy group, the highest scores were related to spiritual growth, nutrition and interpersonal relationship, respectively. In this regard, although the score of spiritual growth was higher among women with gestational hypertension than healthy women, but this difference was not significant [26.53 (3.80) vs 25.81 (3.89), *p* = 0.129]. Also, there was no significant difference in the mean score of interpersonal relationship in both groups. However, the mean score of nutrition-related healthy behaviors were significantly higher in healthy women than in women with gestational hypertension [24.28 (5.02) vs 22.17 (4.45), *p* <0.001]. The lowest scores were related to the dimensions of physical activity, stress management and health, respectively, and the mean score of health responsibility in the group of healthy women was significantly higher than the group of women with gestational hypertension. However, the mean scores dimensions of physical activity and stress management in the two groups were not statistically significant Table 2.

**Table 2:** Comparison of total and sub-scales of main variable scores in two groups

Variable		All ***n***= 300	Pregnancy with induced hypertension ***n*** =100 Mean(SD)	Healthy pregnant women ***n***= 200 Mean(SD)	***P***-Value^**a**^
Interpersonal relationship		23.66(3.67)	23.15(3.27)	23.91(3.84)	0.092
Health responsibility		21.89(4.68)	20.60(4.70)	22.53(3.80)	0.001^*^
Physical activity		17.46(4.09)	17.29(3.99)	17.81(4.29)	0.301
Spiritual growth		26.05(3.87)	26.53(3.80)	25.81(3.89)	0.129
Nutrition		23.58(4.93)	22.17(4.45)	24.28(5.02)	<0.001^*^
Stress management		20.38(4.15)	20.45(4.12)	20.24(4.21)	0.688
**Total score of HPLP-II**		132.71(17.89)	129.83(15.04)	134.15(19.03)	0.049^*^
**Self-Efficacy**	**Mean(SD)**	54.16(6.75)	53.98(6.58)	54.25(6.85)	0.749
**Total Score of Social Support**	**Mean(SD)**	63.70(12.29)	60.14(13.13)	65.49(11.47)	<0.001^*^
**Level of social support**	**n (%)**				
Low social support		37(12.3)	23(7.7)	14(4.7)	
Moderate social support		150(50.0)	48(16.0)	102(34.0)	
High social support		113(37.7)	29(9.7)	84(28.0)	
Social support form specific people	**Mean(SD)**	23.20(4.17)	22.65(4.44)	23.47(4.02)	0.109
Friend	**Mean(SD)**	17.32(6.76)	15.93(7.20)	18.02(6.44)	0.012^*^
Family	**Mean(SD)**	23.19(4.22)	21.56(4.59)	24.01(3.77)	<0.001^*^
**perceived stress**	**Mean(SD)**	39.78(7.79)	44.30(7.84)	37.53(6.73)	<0.001^*^
**Level of perceived stress**	**n(%)**				
low		43(14.3)	6(14.0)	37(12.3)	
High		257(85.7)	94(36.6)	163(66.7)	

^a^ Mann-Whitney U, ^*^ statistically significant

### Comparison of intermediary social determinants of health between two groups

First, the mean score of self-efficacy in healthy pregnant women was higher than pregnant women with gestational hypertension, but there was no statistically significant difference [54.25 (6.85) vs 53.98 (6.58), *p* = 0.749]. Second, a comparison of social support and perceived stress in the case and control groups showed that the total score of social support in healthy women was significantly higher than women with gestational hypertension [65.49 (11.47) vs 60.14 (13.13), *P* < 0.001]. The majority of participants in both groups had moderate perceived social support (34% vs. 16%). However, in healthy pregnant women, the highest score was related to family social support, and the difference between the two groups was statistically significant. While in pregnant women with hypertension, the highest score was obtained in the field of social support from others, however, this difference between the two groups was not statistically significant. Finally, the comparison of the mean score of perceived stress between the two groups showed that the perceived stress in women with gestational hypertension was significantly higher than healthy group [44.30 (7.84) vs 37.53 (6.73), *P* <0.001]. However, most participants in both groups had higher levels of perceived stress (36.6% and 66.7%, respectively) Table 2.

### Factors associated with gestational hypertension

The association between socio-demographic, health-promoting behavior, and psychosocial characteristics with gestational hypertension were assessed. In the multivariate analysis, those women with high stress had about 1.13 times higher odds of gestational hypertension than women who haven’t [AOR 1.13, 95% CI (1.08–1.18)]. Likewise, those pregnant women whose Spouse’s Educational status was low had 4.94 times higher odds of gestational hypertension than women who haven’t [AOR 4.94, 95% CI (1.54–15.81)]. Also, the development of gestational hypertension was decreased by increasing the score of social support [AOR 0.96, 95% CI (0.93–0.98)]. Although the total score of health-promotion behaviors [AOR 0.992, 95% CI (0.98–1.02), self-Efficacy [AOR 0.99, 95% CI (0.95–1.04), and housing [AOR 0.61, 95% CI (0.34–1.07) were protective against hypertension, were not significant Table 3.

**Table 3:** A association between variables with hypertension in pregnant women using multivariate logistic regression analysis

Predictors	B	SE	OR	95% CI	Wald	*P*-Value
Model 1						
Total perceived stress	0.129	0.022	1.137	1.089, 1.188	34.106	<0.001
Total Social Support	-0.040	0.013	0.961	0.936,0.986	8.949	0.003
Total Self-Efficacy	-0.005	0.023	0.995	0.952, 1.040	0.043	0.837
Total Health-promoting	-0.004	0.010	0.992	0.986, 1.023	0.202	0.653
Spouse’s Educational status					9.525	0.023
Secondary school(1)	1.599	0.593	4.948	1.548, 15.815	7.273	0.007
Diploma(2)	0.978	0.425	2.660	1.157, 6.117	5.305	0.021
University(3)	1.049	0.412	2.854	1.273, 6.398	6.485	0.011
Housing N0(1)	-0.495	0.288	0.610	0.347, 1.073	2.947	0.086

*OR*Odd Ratio, *CI* Confidence Interval, *S. E* Standard Error, *B* unstandardized coefficient

### Association between health-promoting behaviors with intermediary social determinants of health

The results of Spearman correlation test showed that there was a significant and positive association between health-promoting behaviors with social support (*r* = 0.427, *p* <0.001, Medium effect) and self-efficacy (*r* = 0.246, *p* = 0.001, small effect). There were significant and negative association between health-promoting behaviors with perceived stress (*r* = - 0.185, *p* = 0.001, small effect). Also, social support had a significant and positive association with self-efficacy (*r* = 0.184, *p* <0.001, small effect) and a significant and negative association with stress (*r* = -0.113, *p* = 0.04, small effect).

First of all, by using univariate linear regression, the effect of social support, self-efficacy and perceived stress variables was examined separately with the total mean score of HPLP in models 1, 2 and 3. The results showed that the independent variables were able to explain 16%, 7.5% and 2.3% of changes in health-promoting behaviors, respectively. In other words, by increasing one standard deviation in the variables of social support and self-efficacy, the score of health-promoting behaviors increases by 0.404 and 0.284 standard deviation, respectively. But by increasing one standard deviation in the perceived stress variable, the score of HPLP decreases by 0.162 standard deviation.

Then, in model 4, using multivariate linear regression, the variables of social support, self-efficacy and perceived stress were entered into the model simultaneously, which accounted for 21.2% of the total changes in the score of HPLP (R^2^adj = 0.212). In this model, social support and perceived stress had the highest (β = 0.348) and lowest (β = -0.114) regression effects on the score of health-promoting behaviors, respectively.

Finally, in regression model 5, the education variable that had a significant association with the dependent variable along with the three independent variables of the research were entered into the model. The results of this model showed that 21.8% of the total changes in the score of health-promoting behaviors depend on these 4 variables (R^2^adj = 0.218). The results also showed that the two variables of social support and self-efficacy have the most effect, respectively and the education variable has the least effect on the score of the dependent variable. Thus, by increasing a standard deviation in the variables of social support, self-efficacy and education, the score of health promotion behaviors will increase by 0.331, 0.215 and 0.094 standard deviation, respectively. In this regard, the perceived stress variable has a negative effect on the dependent variable and by increasing a standard deviation in stress, health-promoting behaviors decrease by 0.112 standard deviation Table 4.

**Table 4:** Results of Hierarchical Regression Analyses Regressing HPLP-II during pregnancy

Predictors	Unstandardized Coefficient, *β*	SE	Standardized Coefficient, *β*	95% CI	R^2^	Adjusted R^2^	F	P-Value
Model 1								
Social Support	0.004	0.001	0.404	0.003 to 0.006	0.163	0.160	57.984	<0.001
Model 2								
Self-Efficacy	0.006	0.001	0.284	0.004 to 0.088	0.081	0.075	26.202	<0.001
Model 3								
perceived stress	-0.003	0.001	-0.162	-0.005 to -0.001	0.026	0.023	8.033	0.005
Model 4								
Self-Efficacy	0.004	0.001	0.215	0.002 to 0.006	0.220	0.212	27.866	<0.001
Social Support	0.004	0.001	0.348	0.003 to 0.005				
perceived stress	-0.002	0.001	-0.114	-0.004 t0 0.000				
Model 5								
Self-Efficacy	0.004	0.001	0.215	0.002 to 0.006	0.229	0.218	21.866	<0.001
Social Support	0.004	0.001	0.331	0.003 to 0.005				
perceived stress	-0.002	0.001	-0.112	-0.004 to 0.000				
Mother’s Educational status	0.017	0.009	0.094	-0.002 to 0.035				

## Discussion

Pregnancy-induced hypertension is one of the major problems in pregnancy that can affect pregnancy outcomes. In this paper, we assessed the comparison between health-promoting behavior and psychological factors in pregnant women with gestational hypertension and without it and Predictors factors for gestational hypertension. Also, we examined the factors which affect health-promoting lifestyle. The results of the study revealed that health-promoting behaviors, perceived social support, and stress were different between the two groups, however, there was no difference in self-efficacy between the two groups. Perceived stress and social support can lead to increasing and decreasing the odds ratio of gestational hypertension, respectively. As well as, social support and self-efficacy were positively correlated with health-promoting behaviors but at the same time, perceived stress had a negative correlation.

Studies on performance of healthy behaviors in pregnant women showed similar results in different countries [2, 22]. Only in two studies from Thailand [43] and Turkey [44], a better overall score is reported. This difference can be attributed to the cultural differences and gestational age of study participants, because they studied pregnant women in their second trimester, which women have more stable condition than their third trimester. The difference between scores of two groups were important and the results are comparable with other studies in such a way that in some studies, obese women and overweight women as high-risk pregnancies had lower scores compared to the comparison groups [7, 45]. Studies on women with preeclampsia also indicate that health promoting life style behaviors is not so suit in these women [2]. In other studies, pregnant women with diabetes [46], women under the age of 18 years [47], and low-income pregnant women [48] had poorer caring behaviors than low-risk groups.

In terms of various aspects of health-promoting lifestyle behaviors score, our results were in the same direction with other studies [6, 49, 50]. So that in these studies, the highest scores were related to self-actualization, nutritional status and social support domains, intermediate scores for responsibility and lowest scores were related to physical activity and stress management, respectively. The results showed that health-promoting behaviors, although it could have a protective effect on gestational hypertension, but this effect was not significant [CI95% (0.986, 1.023)]. However, it should be noted that for participants in the case group, scores earned in all categories were lower than the control group, particularly the nutritional status and health responsibility in pregnant women with hypertension. This is consistent with the study of Momeni-javid et al., 2015 [46], which compares life style promoting behaviors in two groups of healthy and diabetic women. The results showed involved women had a higher body mass index than the control group, which could represent inappropriate nutrition. The results also in line with the results of our previous study on pregnant women [7, 51]. While the majority of pregnant mothers want to change their behavior and ability to provide healthy food and physical activity, many women believe such changes are outside their control [20], according to other researchers. The major obstacles cited by pregnant mothers include lack of adequate opportunity [20, 21] existence of pregnancy complications, lack of awareness and family support, mood changes and fatigue during pregnancy [20].

Self-efficacy was compared in two groups of healthy and hypertensive pregnant women. Although healthy pregnant women acquired higher scores in terms of self-efficacy, there was no significant difference between the two groups gained moderate scores such as in other studies [52–54]. The results showed a protective effect of self-efficacy on hypertension, but it was not significant. According to researchers, interventional programs to increase the self-efficacy of pregnant women can increase their participation in self-care programs and healthy lifestyles. As a result, the consequences of pregnancy can be improved [55, 56]. In the study of Xiaoyan et al., [57] a positive relationship was found between self-efficacy and increased awareness of the disease and the ability to manage the disease. In addition, according to researchers, self-efficacy level is an optimistic or pessimistic picture of patients before starting each function and individuals with higher levels of self-efficacy have more effort to resolve their problems than individuals with lower self-efficacy [58]. Also, the relationship between self-efficacy and social support have been observed that both of them can improve the woman’s ability to cope with the problematic situation [59, 60]. A study by Homko et al. Showed that interventions based on self-efficacy were able to increase women's empowerment to control their diabetes [61].

The comparison of the social support situation in two groups indicated that the degree of understanding social support in both groups was moderate. The results revealed a protective effect of social support on hypertension and women who had a high sense of social support had a lower odds ratio of developing hypertension. However, women with pregnancy hypertension had significantly lower scores, especially the scores for family and friends support domains were below the control group scores. Social support is an important factor in addressing health behaviors and reducing the stress of individuals. Particularly when a woman is subject to high-risk pregnancy, social support to cope with stressors can play a key role [62] and a contributing factor for promoting physical and mental health . Most studies also indicate an average score for perceived social support in pregnant women [22, 63, 64], in line with the present study. Also in different studies on pregnant women including women with diabetes [46], and unwanted pregnancy [65], the degree of understanding social support was less than the control group, which is consistent with our study. According to researchers, receiving family support, especially from husband in women with high-risk pregnancy, was effective in improving psychological performance and could lead to improved relations and quality of life [66]. As mentioned social support can improve self-efficacy and women who simply receive more advocacy and support from their partner, family and friends may have a better status to cope with pregnancy. So, the pregnant woman can engage in the healthy lifestyle program.

As it was predictable, perceived stress in women with hypertension was higher than the control group. And higher levels of perceived stress increase the chance of developing hypertension by 13%. The previous observations indicated high levels of stress in women with preeclampsia as well [62]. Nevertheless, there was no relationship between perceived stress and the risk of diabetes [46, 67]. According to reports, stressful situation, with direct effect on the axis of hypothalamus, pituitary and adrenal, increases cortisol levels, which is associated with cellular immunity, endothelial dysfunction and hypertension [62]. Another important point is that the majority of participants in both groups reported high levels of stress. In previous studies, the stress level in pregnant women was moderate or high. Especially the amount of stress was increased in the third trimester of pregnancy [68]. These results consistent with our study. Not only pregnancy is stressful but also factors like socio-economic status and being in a high-risk condition will increase stress levels. This indicates that women need to receive protection and educational supports to overcome stressors [46].

Data analysis showed a positive association between health-promoting lifestyle with self-efficacy and social support, also negative association was seen between health-promoting lifestyle and perceived stress. According to studies on women who have given birth, social support was a predictor for all domains of health-promoting behaviors excepting physical activity [69]. In other studies, the relationship between social support [63, 70, 71], and self-efficacy with healthy lifestyle is considered [54, 72]. Also, high - risk behaviors and reducing healthy behaviors were noted in the form of stress in previous studies. Obtaining scores of severe stress in pregnant mothers is important. More than half of the participants (n=150) reported severe and very severe stress that required further studies and related interventions. It is obvious and other researchers have also reported that having a healthy lifestyle is a preventing factor to halt undesirable pregnancy outcomes [73]. So as healthy lifestyle interventions were cost-effective [74].

### Limitations

This research consisted of potential restrictions, which is necessary to be considered at the time of data interpretation: 1. Convenient sampling without matching was performed. 2. Due to the type of sampling, socio-demographic variables such as educational and income status have different distributions between cases and controls, which can be considered one of the limitations of the study. 3. In present study, like observational studies, the findings were modified by the confounders; therefore, it is impossible to deny and overlook these factors.

## Conclusion

Women with gestational hypertension have unhealthier lifestyles and having a high level of stress is a risk factor for gestational hypertension but social support has a protective effect on it. In addition, social support status, perceived stress, and self-efficacy can affect health-promoting behaviors. Recognizing the risk factors of gestational hypertension could help the determination of high-risk cases and it is important to pay attention to women's psychosocial factors to create appropriate sources of social support and provide the necessary action to reduce stress. It is essential to establish a group or individual counseling and provide the necessary support for reducing stress in pregnant women. As well, the pay attention to self-efficacy could lead to adopting a healthy lifestyle and preventing illnesses, and reducing undesirable consequences of pregnancy.

## Data Availability

The datasets used and/or analyzed during the current study are available from the corresponding author on reasonable request.

## Abbreviations

HPLP: Health-Promoting Lifestyle Profile
PSS: Perceived Stress Scale
